# Modified reverse-puncture anastomotic technique vs. traditional technique for total minimally invasive Ivor-Lewis esophagectomy

**DOI:** 10.1186/s12957-020-02093-2

**Published:** 2020-12-09

**Authors:** Xiaokang Shen, Tianming Chen, Xiaoming Shi, Ming Zheng, Zhang Yan Zhou, Hai Tao Qiu, Jiawei Zhao, Peng Lu, Po Yang, Shilin Chen

**Affiliations:** 1 Department of Thoracic Surgery, Nanjing Medical University Affiliated Cancer Hospital, Jiangsu Cancer Hospital, Jiangsu Institute of Cancer Research, Baiziting 42, Xuanwu District, Nanjing, 210009 Jiangsu China; 2 Department of Surgery, The Affiliated Hospital of Guizhou Medical University, Guiyang, 550025 China; 3 Department of Cardiothoracic Surgery, Nanjing Medical University Third Affiliated Hospital, Sir Run Run Hospital, Nanjing Medical University, Nanjing, 211100 China; 4 Department of Thoracic Surgery, Taikang Xianlin Drum Hospital Affiliated to Medical College of Nanjing University, Nanjing, 210046 China; 5 School of Life Science, Nanjing Normal University, Nanjing, 210046 Jiangsu China

**Keywords:** Modified reverse-puncture anastomotic technique, Total endoscopic Ivor-Lewis esophagectomy, Medial and lower esophageal cancer

## Abstract

**Background:**

Total endoscopic Ivor-Lewis esophagectomy is a challenging, complex, and costly operation. These disadvantages restrict its wide application. The aim of this study was to compare the modified reverse-puncture anastomotic technique and traditional technique for total minimally invasive Ivor-Lewis esophagectomy.

**Methods:**

In this cohort retrospective study, all patients with medial and lower squamous cell carcinoma of esophagus from February 2014 and June 2018 were divided into two groups according to the surgical method, which were modified reverse-puncture anastomotic technique group and traditional technique group. The operation time, intraoperative bleeding volume, complications, and cost of the two groups were compared.

**Results:**

Forty-eight patients in the modified reverse-puncture anastomotic technique group while 54 patients in the traditional technique group were included. The operation time was 293.4 ± 57.2 min in the modified reverse-puncture anastomotic technique group, which was significantly shorter than that in the traditional technique group (353.4 ± 64.1 min) (*P* < 0.05). The intraoperative bleeding volume of modified reverse-puncture anastomotic technique group was 157.3 ± 107.4 ml, while it was 191.9 ± 123.6 ml in traditional technique group (*P* = 0.14). There were similar complications between the two groups. The cost of modified reverse-puncture anastomotic and traditional technique in our hospital were and 72 ± 13 and 83 ± 41 thousand Yuan, respectively (*P* = 0.08).

**Conclusion:**

The good short-term outcomes that were achieved suggested that the use of modified reverse-puncture anastomotic technique is safe and feasible for total endoscopic Ivor-Lewis esophagectomy.

## Introduction

It is reported that the esophageal cancer ranks the third of incidence of malignant tumors and fourth of mortality in China, which seriously threatens the health of the Chinese people [1]. At present, the treatment of esophageal cancer includes surgical resection, radiotherapy and chemotherapy, targeted therapy, and so on [2]. Surgical resection is still the preferred treatment. Ivor-Lewis esophagectomy is a classic procedure for medial and lower esophageal cancer, which effectively avoids the defect of cervical anastomosis [3]. However, the thoracic anastomosis of esophagus and stomach in total endoscopic Ivor-Lewis esophagectomy still has some problems, such as technical difficulties, complicated operation, and high cost, which lead to little development at home and abroad [2, 3]. Modified reverse-puncture anastomotic technique is based on a new idea for completely intracorporeal anastomosis combined with transanal specimen extraction, and the procedure can be easily performed. This technique has more commonly been reported to be applied in laparoscopic gastric cancer surgery [4, 5]. Modified reverse-puncture intrathoracic anastomosis with common stapler under complete thoracoscopy is rarely reported. This study was to compare the modified reverse-puncture anastomotic technique and traditional technique for total minimally invasive Ivor-Lewis esophagectomy. In this report, based on our experience in 48 total endoscopic Ivor-Lewis esophagectomy, we described a thoracoscopic anastomosis using a common stapler with reverse-puncture anastomosis technique, which achieved good results.

## Methods

### Patients

The patients with medial and lower squamous cell carcinoma of esophagus in the Department of Cardiothoracic Surgery, who underwent total endoscopic Ivor-Lewis esophagectomy for cancer between February 2014 and June 2018, were retrospectively analyzed. All patients were divided into two groups according to the surgical method, which were modified reverse-puncture anastomotic technique group and traditional technique group. This study was approved by the applicable institutional review board of Nanjing Medical University Third Affiliated Hospital.

#### Inclusion criteria

(1) Patients were preoperatively diagnosed with medial and lower squamous cell carcinoma of esophagus by gastroscopy and biopsy. (2) Preoperative pathological diagnosis was squamous cell carcinoma by electronic gastroscope. The invasion of the cancer was not obvious. The lymph nodes of abdominal cavity and mediastinum were less than 2 cm, and the lymph nodes of neck were not enlarged. (3) Head CT, bone scan, and abdominal B-ultrasonography suggest that there is no distant metastasis of cancer.

#### Exclusion criteria

(1) Patients had severe comorbidities such as impaired cardiac, kidney, liver, and/or lung function. (2) Patients had tumors staged above T4b or M1 based on preoperative evaluation. (3) The patient had severe chest adhesions and had a history of right chest trauma, surgery, and tuberculosis.

### Anesthesia

All patients received intravenous combined general anesthesia, double-lumen endotracheal intubation (or single-lumen endotracheal intubation plus occluder). Bilateral lung ventilation was used for laparoscopic surgery and left one lung ventilation was used for thoracoscopic surgery.

### Surgical technique

#### Abdominal operation

**Fig. 1:**
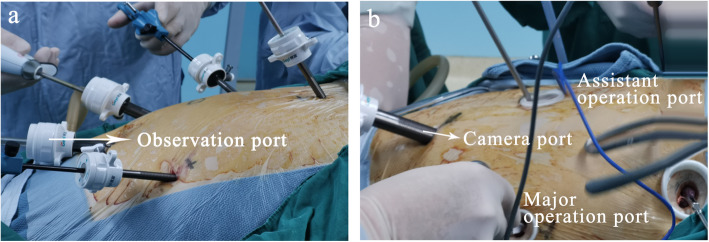
**a** Five-hole method. **b** Four-hole method

The patients were taken to supine positions with head and right elevations of 15–30°. Artificial pneumoperitoneum (1.176–1.372 kPa) was established by CO_2_ through subumbilical puncture hole (10 mm trocar observation hole). “Five-hole method” programmed free gastric body (Fig. 1a). Twelve millimeters trocar under the umbilicus was placed into the laparoscope as an observation hole. One observation hole was placed beside the navel of bilateral clavicle midline (right 12 mm, as main operation hole which was placed into the ultrasonic scalpel; left 5 mm, as assistant operation hole). Five millimeters trocar under right costal arch in clavicle midline was as assistant hole and 24 cm below xiphoid process was as 5 mm trocar for liver traction (fix the right diaphragmatic foot with a dental forcep without assistant pulling). The presence or absence of abdominal adhesions and metastasis was explored. Ultrasound scalpel was used to separate the omental tissue greater curvature along the lateral side of gastroepiploic vascular arch. The gastrointestinal ligament, splenogastric ligament, short gastric vessel, and pericardial vessel were separated, and cardiac lymph nodes were cleared. Pull the left lobe of the liver, open the small omental sac, disconnect the ligament of the liver and stomach, and keep the right gastric vessel. The stomach was lifted to the upper left, three branches of the celiac trunk were exposed and skeletonized, and lymph nodes beside the common hepatic artery, splenic artery, and left gastric artery were cleaned. Double-clamp the left gastric artery with a Hem-OLock clip and disconnect it. The small curved gastric vessels were treated at 35 cm above the pylorus and most of the tubular stomach was made with 3-4 Echelon 60. Finally, the left and right diaphragm feet were exposed and the esophageal hiatus (5 cm) was enlarged. The stomach was placed in the normal anatomical position and the abdominal cavity latex tube was placed routinely.

#### Thoracoscopic operation

**Fig. 2:**
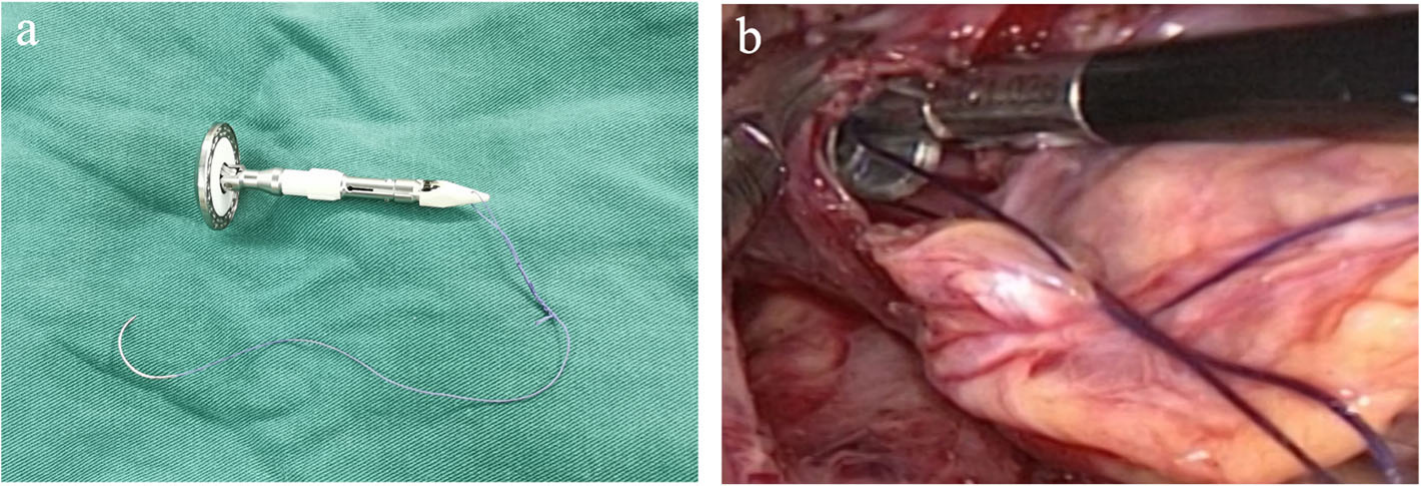
**a** The reserved puncture device was made by extending the anvil belt of the base nail pedestal of the circular stapler of the Qiansheng digestive tract. **b** The reverse-puncture device was pushed to the neck along the long axis of the esophagus to insert the reverse-puncture device

The patient was reverted to the left semi-prone position and left one lung was ventilated. “Four-hole method” was performed (Fig. 1b): The main operating hole was located in the fifth intercostal space of the anterior axillary line (about 1.5 cm); laparoscopic hole was located in the 8th intercostal space of the posterior axillary line (about 1.5 cm). The two auxiliary operating holes are located in the third intercostal space of the anterior axillary line and the seventh intercostal space of the scapula line (about 1 cm). The mediastinal pleura was opened under thoracoscopy. Hem-OLock was used to cut off the azygos arch; then, the tumors and esophagus were freed by electrocautery or ultrasonic scalpel (assisted by hand if necessary). At the same time, lymph nodes in inferior pulmonary ligament, paraesophagus, subcarina of trachea, right and left main bronchus, upper mediastinum, and right and left recurrent laryngeal nerve chain were dissected. The reserved puncture device was made by extending the anvil belt of the base nail pedestal of the circular stapler (Ethicon, Johnson & Johnson, New York, NY, US) and connecting the traction line from its side hole in modified reverse-puncture anastomotic technique group (Fig. 2a).

Appropriately enlarge the main operating hole to a diameter of about 3 cm, and use an incision protector to facilitate the placement of the stapler abutment and the anvil head. The incised esophageal cavity was exposed with endoscopic forceps and aspirators, and the reverse-puncture device was pushed to the neck along the long axis of the esophagus to insert the reverse-puncture device in modified reverse-puncture anastomotic technique group (Fig. 2b).

**Fig. 3:**
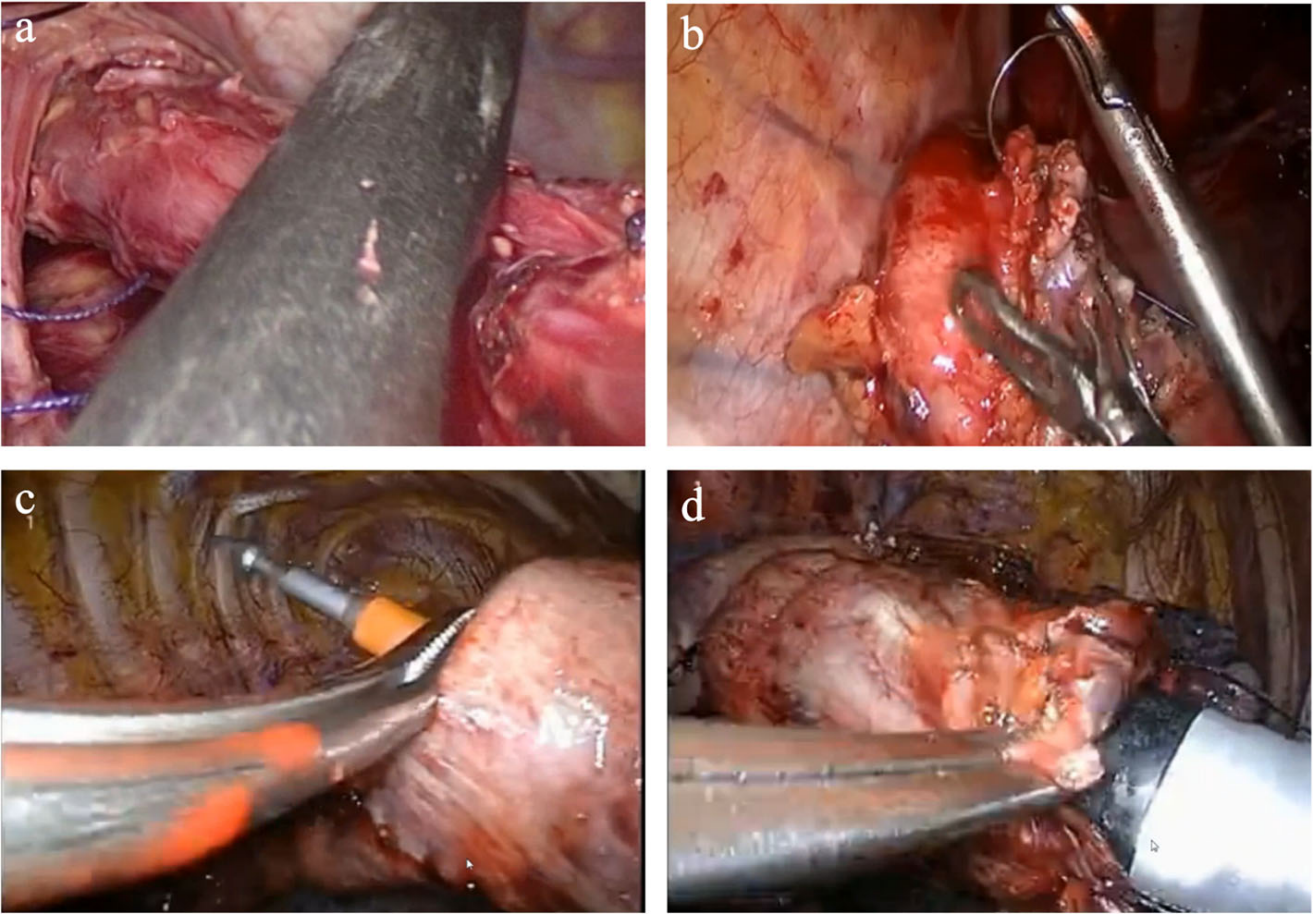
**a** Use the oval forceps to pull the esophagus down slightly, and use the Johnson rotating gold nail to cut the esophagus, leave a 2-mm-diameter hole in the left anterior-lower corner of the esophageal stump and pull out the traction line. **b** The control line for U-shaped suture of tubular stomach was pierced through the main operating hole. **c** The stapler body was put into the stomach through the main operating hole. **d** Complete the end-to-side anastomosis between the tubular stomach and the esophagus

Use the oval forceps to pull the esophagus down slightly, and use the Johnson rotating gold nail (ENDO-GIA 60-1.8) to cut the esophagus above the tumor at a 45° angle, leave a 2-mm-diameter hole in the left anterior-lower corner of the esophageal stump and pull out the traction line (Fig. 3a). Then, the small hole was appropriately enlarged by an ultrasonic knife or an electric hook, and the anvil of the nail base was taken out to completely expose the esophageal stump, and the traction line was cut.

The tubular stomach was lifted from the enlarged esophageal hiatus to the thoracic cavity. At the end of the last shot of the tubular stomach, 1.0 cm near the cardia, the lesser curvature side of the gastric wall was cut about 3 cm, and the control line for U-shaped suture of tubular stomach was pierced through the main operating hole (Fig. 3b).

**Fig. 4:**
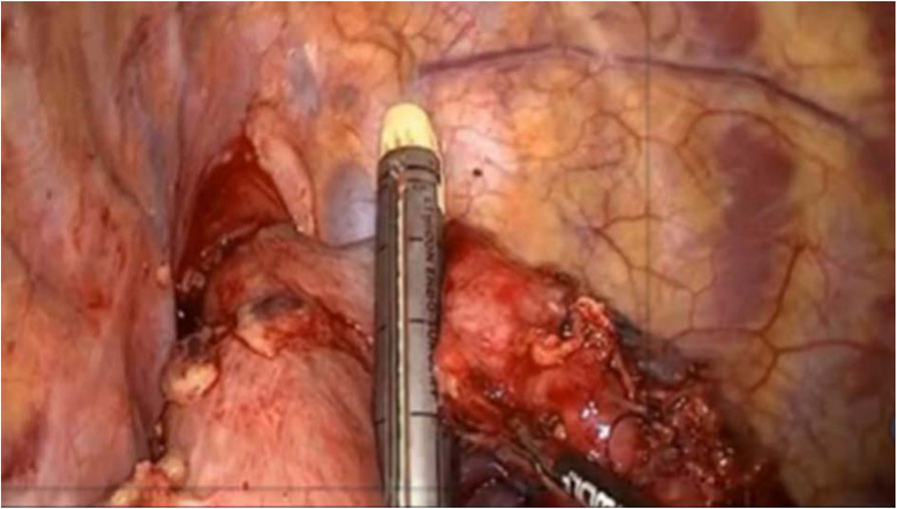
Tumors of the middle and lower esophagus, cardia, and lesser gastric curvature lymph nodes was cut off using 1–2 60 rotating blue nails

The stapler body was put into the stomach through the main operating hole, and the tubular stomach was suitably pulled, and the center rod at the top of the stapler was rotated to pass it out from the stomach wall at a distance of 3.0 cm from the traction line hole at the head of the tubular stomach (Fig. 3c). Then, the central rod was rotated and the stapler was activated to complete the end-to-side anastomosis between the tubular stomach and the esophagus (Fig. 3d). After the anastomosis was completed, the endoscope was used as an “endoscope”, and the anastomosis was checked into the tubular stomach to ensure that the anastomosis was complete without bleeding. Tumor resection of the middle and lower esophagus, cardia, and lesser gastric curvature lymph nodes was performed with 1–2 60 rotating blue nails from the stump of the tubular stomach to 1 cm above His angle of the greater gastric curvature (Fig. 4). A non-invasive absorbable silk thread was used to suture and knot the mediastinal pleura-proximal tubular gastric head, the mediastinal pleura-tubular gastric wall, the parietal pleura-tubular gastric wall with one needle to complete the relaxation and immobilization anastomosis, and the gastrointestinal decompression tube was inserted and two thoracic tubes were routinely placed for drainage.

### Postoperative management

The thoracic, abdominal, and gastric canals should be drained smoothly in the early stage after operation. Strengthen the management of respiratory tract and encourage early cough and expectoration. Enteral nutrition can be started on the second day after operation. Upper digestive tract iodine water radiography was performed on the seventh day after operation to observe anastomotic stoma and gastric emptying and then gradually began to eat through the mouth and flow to the general diet.

### Outcomes

The operation time, intraoperative bleeding volume, complications, and cost were recorded by the clinician.

### Statistical analysis

All data were expressed in the form of mean ± standard deviation (SD). Comparison between the two groups was conducted through two-tail *t* test, and multiple sets of data (> 2) were compared through the one-way ANOVA, with *p* < 0.05 meaning statistical significance. All data were analyzed with the SPSS 23.0.

## Results

### Basic characteristics

Medical records from 48 patients (34 males and 14 females) with medial and lower esophageal cancer were retrospectively included in modified reverse-puncture anastomotic technique group. The average age of the patients was 54 ± 6.5 years old (47–72 years old). The tumors were located at the middle and lower esophagus, 31 in the middle thoracic esophageal cancer and 17 in the lower thoracic esophageal cancer. Fifty-four patients (38 males and 16 females) with medial and lower esophageal cancer were retrospectively included in traditional technique group. The average age of the patients was 56 ± 7.1 years old (43–75 years old). The tumors were located at the middle and lower esophagus, 41 in the middle thoracic esophageal cancer and 13 in the lower thoracic esophageal cancer. There were no statistical differences of the basic characteristics between the two groups (*P* > 0.05) (Table 1).

**Table 1:** Basic characteristics

	Modified reverse-puncture anastomotic technique group	Traditional technique group	
Patients (*n* (male))	48 (34)	54 (38)	*P* > 0.05
Age (range) (years)	54 ± 6.5 (47–72)	56 ± 7.1 (43–75)	*P* > 0.05
Middle thoracic esophageal cancer	31	41	*P* > 0.05
Lower thoracic esophageal cancer	17	13	*P* > 0.05

### Operation time and the length of hospitalization

All patients underwent anastomosis under endoscopy and the operation was smooth without auxiliary incision in the chest and abdomen and conversion to thoracotomy.

The operation time was 293.4 ± 57.2 min in modified reverse-puncture anastomotic technique group, which was significantly shorter than that in traditional technique group (353.4 ± 64.1 min) (*P* < 0.05). The thoracoscopic operation time was 195.6 ± 64.5 min in modified reverse-puncture anastomotic technique group, which was also shorter than that in traditional technique group (249.4 ± 71.8 min) (*P* < 0.05).

Among the 48 patients of modified reverse-puncture anastomotic technique group, 47 had 7.2 ± 1.3 days of parenteral nutrition and 10–16 days (an average of 11 days) of hospitalization after operation. In traditional technique group, 48 patients had 7.3 ± 2.1 days of feeding and 11–17 days (an average of 12 days) of hospitalization after operation (Table 2).

**Table 2:** Operation time and the length of hospitalization

	Modified reverse-puncture anastomotic technique group	Traditional technique group	
Operation time (min)	293.4 ± 57.2	353.4 ± 64.1	*P* < 0.05
Thoracoscopic operation time (min)	195.6 ± 64.5	249.4 ± 71.8	*P* < 0.05
Parenteral nutrition (days)	7.2 ± 1.3	7.3 ± 2.1	*P* > 0.05
Hospitalization (average ± standard deviation (range)) (days)	11 ± 3.2 (10–16)	12 ± 3.5 (11–17)	*P* > 0.05

### Bleeding and drainage

The intraoperative bleeding volume of modified reverse-puncture anastomotic technique group was 157.3 ± 107.4 ml, while it was 191.9 ± 123.6 ml in traditional technique group (*P* = 0.14). The resected lymph node was 15.2 ± 2.5 and 14.4 ± 1.6 in modified reverse-puncture anastomotic technique group and traditional technique group, respectively (*P* = 1.00).

Postoperative pathology showed esophageal squamous cell carcinoma, without cancer involvement at the upper and lower incision margins. The pathological stage of cancer included 7 cases of stage I, 14 cases of stage II, and 27 cases of stage III (20 cases of stage IIIa and 7 cases of stage IIIb) in modified reverse-puncture anastomotic technique group. The pathological stage of cancer included 6 cases of stage I, 17 cases of stage II, and 31 cases of stage III (23 cases of stage IIIa and 8 cases of stage IIIb) in traditional technique group.

The average thoracic drainage volume in the first 3 days after operation was 240, 150, and 110 mL, respectively, in modified reverse-puncture anastomotic technique group and the closed thoracic drainage tube was removed 3 to 12 days after operation, with an average of 5 days. In traditional technique group, the average thoracic drainage volume in the first 3 days after operation was 310, 210, and 160 mL, respectively. Closed thoracic drainage tube was removed 5 to 14 days after operation, with an average of 7 days (Table 3).

**Table 3:** Bleeding and drainage

	Modified reverse-puncture anastomotic technique group	Traditional technique group	
Intraoperative bleeding volume (ml)	157.3 ± 107.4	191.9 ± 123.6	*P* > 0.05
Resected lymph node (*n*)	15.2 ± 2.5	14.4 ± 1.6	*P* > 0.05
The pathological stage of esophageal squamous cell carcinoma			
Stage I	7	6	
Stage II	14	17	
Stage III	27 (20 IIIa and 7 IIIb)	31 (23 IIIa and 8 IIIb)	
Thoracic drainage volume (ml)			
Day 1	240	310	
Day 2	150	210	
Day 3	110	160	
Thoracic drainage tube time (range) (days)	5 (3–12)	7 (5–14)	

### Complications

In modified reverse-puncture anastomotic technique group, the incidence of anastomotic leakage was 2.08% (1/48). Severe pulmonary infection and anastomotic leakage occurred in a patient with severe obesity, diabetes, and hypertension after operation. After conservative treatment such as fasting, acid suppression, enteral nutrition support, and strengthening anti-infection, the patient was cured 42 days after surgery. Similarly, the incidence of anastomotic stenosis was 6.3% (3/48). Among the 3 patients, the symptoms of the patient with severe anastomotic stenosis were relieved after mechanical dilation of the anastomotic stoma. Besides, no serious chylothorax occurred after operation in all patients. Chylous fluid appeared in 2 patients during enteral nutrition infusion on the second day after operation. The amount of chylous fluid was about 300 ml. With stopping intestine perfusion of any fluid, changing to complete intravenous nutrition support, chylous fluid disappeared after 3 days of treatment with somatostatin. The patient ate normally after a week and did not have chylous fluid again.

In traditional technique group, the incidence of anastomotic leakage was 9.25% (5/54). Severe pulmonary infection and anastomotic leakage post operation occurred in 9 patients with severe obesity, diabetes, and chronic bronchitis or emphysema. After conservative treatment such as fasting, acid suppression, enteral nutrition support, and strengthening anti-infection, the anastomotic leakage was cured 35~58 days after surgery. Similarly, the incidence of anastomotic stenosis was 16.6% (9/54). Among the 9 patients, the symptoms of the 4 patients with severe anastomotic stenosis were relieved after mechanical dilation of the anastomotic stoma. Besides, no serious chylothorax occurred after operation in all patients. Chylous fluid appeared in 1 patient during enteral nutrition infusion on the third day after operation. The amount of chylous fluid was about 500 ml. With stopping intestine perfusion of any fluid, changing to complete intravenous nutrition support, chylous fluid disappeared after 6 days of treatment with somatostatin. The patient ate normally after a week and did not have chylous fluid again.

The incidence of anastomotic leakage of modified reverse-puncture anastomotic technique group showed no significant difference compared with that of traditional technique group (*P* = 0.124), while the incidence of anastomotic stenosis of modified reverse-puncture anastomotic technique group showed no significant difference compared with that of traditional technique group (*P* = 0.103), though both of them showed a decrease trend in modified reverse-puncture anastomotic technique group compared with traditional technique group.

### Treatment cost

The cost of modified reverse-puncture anastomotic and traditional technique in our hospital were and 72 ± 13 and 83 ± 41 thousand Yuan, respectively (*P* = 0.08).

## Discussion

In this study, we have compared the modified reverse-puncture anastomotic technique and traditional technique for total minimally invasive Ivor-Lewis esophagectomy. The operation time, intraoperative bleeding volume, complications, and cost of modified reverse-puncture anastomotic technique was less compared with traditional technique for total minimally invasive Ivor-Lewis esophagectomy.

It was reported for the first time in 2000 that thoracoscopy combined with laparoscopy was used for esophageal cancer surgery [6]. The combination of thoracoscopic and laparoscopic surgery has the advantages of minimal trauma, less impact on respiratory function, and rapid recovery after operation, while maintaining thoracic and abdominal integrity. However, due to the difficulty of inserting stapler’s nail anvil and making purses under endoscopy, most of the previous thoracoscopic esophageal cancer operations were performed thoracic and abdominal operations under thoracoscopy, followed by McKeown operation for cervical incision anastomosis. The main advantage of McKeown surgery is that even if an anastomotic fistula occurs, it is not a fatal complication, and more conservative treatment can be used to heal. However, compared with Ivor-Lewis operation, the procedure is cumbersome, the operation time is long, the trauma is large, and the symptoms of hoarseness, drinking cough, anastomotic fistula, and anastomotic stenosis during perioperative period seriously affect the quality of life of patients, which is contrary to the original intention of minimally invasive surgery [7]. Therefore, it is ideal for the use of minimally invasive Ivor-Lewis esophagectomy (MIILE) for the middle and lower esophageal cancer. However, MIILE is complicated in operation. The biggest technical difficulty lies in how to successfully complete the reconstruction of the digestive tract under thoracoscopy. How to conveniently, quickly, and safely anastomose the gastroesophageal tract is the hotspot for colleagues in thoracic surgery.

At present, there are many anastomotic methods, including manual suture, linear stapler anastomosis, and circular stapler anastomosis. Intrathoracic manual suture has not been popularized due to its complicated operation, high technical requirements, and long operation time [8]. Linear stapler anastomosis, including side-to-side anastomosis and T-shaped anastomosis, does not require intrathoracic nail anvils, but it cannot replace circular stapler anastomosis because of its complicated operation, long anastomotic time, anastomotic leakage, esophageal reflux, and other complications [9–11]. However, the most commonly used round stapler anastomosis is difficult to insert anvil under thoracoscope, which limits its use in MILE. After 2008, it was reported that the use of OrVil technology for the radical treatment of esophageal cancer under the combined thoracoscopic and laparoscopic techniques has achieved good results [5]. OrVil is an integrated device for inserting anvils through the mouth. The system simplifies the process of inserting anvils and thus greatly simplifies the reconstruction of the thoracic digestive tract under total endoscopy. However, this technique will increase the chance of thoracic infection. Its tilted anvil may still be stuck in oropharynx, and even damage oropharynx and upper esophagus. It is also difficult to popularize because of its high price. In this study, the surgical procedure for each patient was determined at the discretion of the operating surgeon considering the affordability of the patients.

So how should we break through the “bottleneck” of the development of minimally invasive surgery for esophageal cancer? Based on the characteristics of OrVil technique, 48 cases of intrathoracic gastroesophageal anastomosis under thoracoscopy were performed using reverse-puncture anastomosis technique with common gastrointestinal anastomotic instruments, which greatly reduced the cost of surgery and achieved satisfactory results. In this study, 48 cases underwent anastomosis under the endoscope, and the operation was smooth. There was no auxiliary incision in the chest and abdomen, and no conversion thoracotomy.

In our study, the cost of modified reverse-puncture anastomotic showed a decreased trend compared with traditional technique, although the difference was not statistically significant. The main reason is that traditional technique uses bag suture instead of rotating gold nail, the cost of surgical instruments is lower than that of modified reverse-puncture anastomotic technique, but the technique of purse string suture under endoscopy is very difficult. Traditional technique takes a long time and has a high failure rate. Many rescue measures are often needed, and it is difficult to ensure the complete docking of the mucosa of esophagogastrostomy. Postoperative complications such as anastomotic leakage, chest and lung infection, and postoperative drainage fluid are frequent. Therefore, the operation time is long, the patient’s internal environment is unbalanced, the anesthesia cost and other additional costs are high, the postoperative protein plasma and nutritional support, the antibiotic application cost is high, the recovery is slow, and the hospital stay time is relatively long.

The security and feasibility of this technology are as follows: (1) The advantage of MIILE is to reduce postoperative complications, but the incidence of major complications after MIILE is still 20.0–36.8% [12, 13]. The incidence of major complications in this study was 8.3% (4/48), which was lower than the reported level. (2) Anastomotic fistula is a common serious complication of esophageal cancer surgery. The incidence of anastomotic fistula in MIILE is about 0–9.1% [13]. Traditionally, intrathoracic anastomotic fistula is more complicated than cervical fistula. It is difficult to deal with and has a high mortality rate, which is also the reason why many surgeons choose McKeown operation. In this study, the incidence of anastomotic leakage was 2.08% (1/48), which was lower than MIILE [12, 13] and open Ivor-Lewis surgery [11]. (3) Anastomotic stenosis is also a common complication after operation, which seriously affects the quality of life of patients after operation. It was reported in the literature that the stenosis rate of traditional circular stapler anastomotic stoma was 22–73%, and 13–40% of patients need multiple dilatation [14]. It is suggested that the diameter of anvil should be increased to prevent anastomotic stenosis [15]. Many studies have shown that anastomotic stenosis is a multifactorial result, which may be related to the anastomotic mode, blood supply and tension, anastomotic fistula, and other comprehensive factors, but not to the diameter of anvil [14]. In this study, 25-mm staplers were used in all patients, and 3 patients (6.3%) needed anastomotic dilatation, which was lower than those reported in the literature. All patients could eat normally after dilatation. (4) Operation time is an important index for evaluating operation methods. The operation time of MIILE was significantly prolonged, ranging from 249.6 to 499.0 min [12, 13, 16]. In this study, the operation time was 293.4 ± 57.2 min, which did not increase significantly compared with the above time. Therefore, it can be concluded that the modified reverse-puncture anastomosis technique is safe, feasible, and more economical for total endoscopic Ivor-Lewis esophagectomy for the treatment of middle and lower esophageal cancer.

There were several previous studies reporting the similar reverse-puncture anastomotic technique for minimally invasive Ivor-Lewis esophagectomy. However, they were all single arm studies. Xiao et al. [17] has included 26 cases and showed that the application of a reverse-puncture anastomotic technique in a minimally invasive Ivor-Lewis esophagectomy can achieve the combined goals of being safe, effective, minimally invasive, and economical. Zhang et al. [18] has reported 15 consecutive patients with cancer in the distal third of the esophagus or the gastric cardia underwent this modified surgical procedure and showed that the good short-term outcomes that were achieved suggest that the modified anastomotic technique is safe and feasible for thoracolaparoscopic Ivor-Lewis esophagectomy.

There were also some limitations in this study. First, this is a retrospective study, prospective study is still needed. Second, this is a study from our one center; further study with multicenter is also needed. Third, in our study, only squamous cell carcinoma was included.

In conclusion, our study described a modified reverse-puncture anastomotic technique for total endoscopic Ivor-Lewis esophagectomy. The operation was smooth and the recovery was fast. Our results found that only one patient with severe obesity, diabetes, and hypertension developed anastomotic leakage after operation due to severe pulmonary infection and four patients had anastomotic stenosis after operation, which showed that this technology worked well. Our results preliminarily indicated that the modified reverse-puncture anastomotic technique for total endoscopic Ivor-Lewis esophagectomy was safe and feasible and had a good short-term effect, but the long-term efficacy needed further follow-up observation. Future studies with large sample size and long-term follow-up are needed to provide a powerful basis for the application of this technology.

## Data Availability

This data and materials are available.

## Abbreviations

MIILE: Minimally invasive Ivor-Lewis esophagectomy
